# Ribotoxic Proteins, Known as Inhibitors of Protein Synthesis, from Mushrooms and Other Fungi According to Endo’s Fragment Detection

**DOI:** 10.3390/toxins14060403

**Published:** 2022-06-13

**Authors:** Nicola Landi, Hafiza Z. F. Hussain, Paolo V. Pedone, Sara Ragucci, Antimo Di Maro

**Affiliations:** Department of Environmental, Biological and Pharmaceutical Sciences and Technologies (DiSTABiF), University of Campania ‘Luigi Vanvitelli’, Via Vivaldi 43, 81100 Caserta, Italy; nicola.landi@unicampania.it (N.L.); hafizazumrafatima.hussain@unicampania.it (H.Z.F.H.); paolov.pedone@unicampania.it (P.V.P.)

**Keywords:** β-fragment, Endo’s assay, enzymes, fungi, mushrooms, N-glycosylases, protein synthesis inhibitors, ribosome inactivating proteins

## Abstract

rRNA N-glycosylases (EC 3.2.2.22) remove a specific adenine (A_4324_, rat 28S rRNA) in the sarcin ricin loop (SRL) involved into ribosome interaction with elongation factors, causing the inhibition of translation, for which they are known as plant ‘ribosome inactivating proteins’ (RIPs). However, protein synthesis inactivation could be the result of other enzymes, which often have rRNA as the target. In this scenario, Endo’s assay is the most used method to detect the enzymes that are able to hydrolyze a phosphodiester bond or cleave a single N-glycosidic bond (rRNA N-glycosylases). Indeed, the detection of a diagnostic fragment from rRNA after enzymatic action, with or without acid aniline, allows one to discriminate between the N-glycosylases or hydrolases, which release the β-fragment after acid aniline treatment or α-fragment without acid aniline treatment, respectively. This assay is of great importance in the mushroom kingdom, considering the presence of enzymes that are able to hydrolyze phosphodiester bonds (e.g., ribonucleases, ribotoxins and ribotoxin-like proteins) or to remove a specific adenine (rRNA N-glycosylases). Thus, here we used the β-fragment experimentally detected by Endo’s assay as a hallmark to revise the literature available on enzymes from mushrooms and other fungi, whose action consists of protein biosynthesis inhibition.

## 1. Introduction

**Figure 1:**
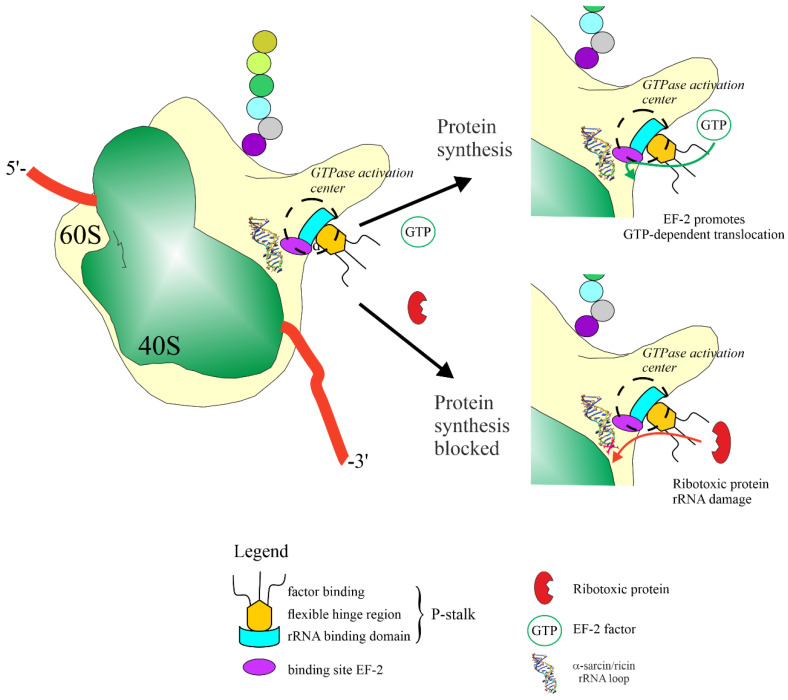
Schematic representation of the ribotoxins and N-glycosylases action on ribosomes during protein synthesis. In absence of enzyme action, the elongation factor is able to recognize the ribosome at the level of SRL (upper panel). The site-specific irreversible modification of SRL caused by the action of a ribotoxic protein (i.e., RIPs, ribotoxins or RL-Ps) prevents translocation, resulting in protein synthesis inhibition (bottom panel). The beginning of both processes consists of EF-2 or ribotoxic protein interaction with P-stalk complex (see main text).

Fungi produce several ribotoxic proteins, known as inhibitors of protein synthesis (i.e., ribonucleases, ribotoxins, ribotoxin-like proteins (RL-Ps) and N-glycosylases), likely as a self-defence mechanism. Despite their broad distribution among these organisms, their biological role is still under consideration. Independently from their action, most of them are site-specific enzymes, which irreversibly modify the same target, i.e., the ribosome, acting on the GAGA tetraloop at the level of single-stranded sarcin ricin loop (SRL) of larger rRNA. This loop is involved in ribosome interaction with prokaryotic or eukaryotic elongation factors (EF-G or EF-2, respectively), by preventing mRNA-tRNA translocation, causing the inhibition of translation and cell death by apoptosis [1]. In particular, the damage to rRNA by ribotoxins and N-glycosylases involves the interaction of these toxins with P-stalk, which is usually necessary to allow the interaction of EF-2-GTP with ribosomes [2]. P-stalk promotes the vicinity of ribotoxic proteins (i.e., N-glycosylase or specific ribonuclease) to SRL, changing the configuration of this loop after site-specific enzymatic action, for which EF-2 is unable to explicate its action (GTP-dependent translocation), as shown in Figure 1 [3,4]. P-stalk is a protein complex that plays a key role in domain-specific recognition of elongation factors in both prokaryotes and eukaryotes, although the organization of P-stalk changes in bacterial, archaeal and eukaryotic ribosomes, due to the diversity of stalk proteins. In addition, this complex improves ribosome GTPase activity when the elongation factors correctly bind to ribosomes, a function that is also depleted when SRL is damaged [5].

The modification of the translocation factors binding site on ribosomes, caused by ribotoxic proteins, can be revealed by the detection of a diagnostic RNA fragment released from rRNA SRL loop. Specifically, ribotoxins from ascomycetes fungi [6,7] and RL-Ps from edible basidiomycetes mushrooms [8,9,10] are specific ribonucleases that catalyze the hydrolysis of a single phosphodiester bond between G_4325_ and A_4326_ of SRL (rat 28S rRNA numbering), releasing a specific rRNA 3′-end fragment called the α-fragment from the molecular mechanism of action of α-sarcin [9,11]. Structurally, ribotoxins and RL-Ps are generally basic proteins with a low molecular weight (range 13–16 kDa) [9,11,12], and show a different catalytic mechanism even if they have the same target (SRL loop). Indeed, the catalytic amino acid residues necessary for ribotoxin activity are two histidinyl residues and a single glutamyl reside (His50 and His137 as well as Glu96, α-sarcin numbering) [11], whereas ribotoxin-like proteins include two aspartyl residues and a single histidinyl residue (Asp68 and Asp70 as well as His77, ageritin numbering) [9].

Conversely, most N-glycosylases (commonly known as plant ‘ribosome inactivating proteins’; RIPs) have been characterized from angiosperms plants (not in gymnosperms), including edible species [13,14,15], while one from algae [16] and a few from bacteria [17] or fungi [15,18,19]. Enzymatically, RIPs are rRNA N-glycosylases (EC 3.2.2.22), being hydrolases (EC 3) acting as glycosylases (EC 3.2) that hydrolyze N-glycosyl compounds (EC 3.2.2). The site-specific rRNA N-glycosylase activity toward ribosomes, due to the recognition of a specific N–C glycosidic bond between an adenine and the nucleotide on SRL of larger rRNA, caused the removal of a specific adenine (A_4324_, rat 28S rRNA numbering), with consequent formation of an abasic (i.e., deadenylated) site. This site is unstable and a reaction of β-elimination can occur after acid aniline RNA treatment, with the consequent cleavage of the rRNA 3′-end fragment, thus called β-fragment [20,21,22]. Furthermore, other enzymatic activities are attributable to rRNA N-glycosylases, such as polynucleotide:adenosine glycosylase (PNAG) on different polynucleotide substrates [23], phosphatase activity on lipids, DNase [24], chitinase and superoxide dismutase actions [20]. Structurally, plant RIPs can be divided into two main groups, depending on the presence or absence of a quaternary structure. Type 1 RIPs (~30-kDa, basic pI) are single chain proteins with enzymatic action, whereas type 2 RIPs (~60-kDa, neutral pI) present a quaternary structure, consisting of an enzymatically active A-chain linked to a B-chain with lectinic properties through a disulphide bridge. The B-chain binds to carbohydrates on the cell surface, allowing A-chain cell internalization. The absence of a lectinic chain prevents type 1 RIPs to bind to the cell, which are consequently less toxic with respect to type 2 RIPs, due to the difficulty of entering the cell [15]. Moreover, a third group of RIPs, known as type 3 RIPs, consists of a type 1-like N-terminal domain with N-glycosylase activity, covalently linked to a C-terminal domain with unknown function [25]. This group includes non-canonical single chain toxins, including b-32 from maize and JIP-60 from barley [26]. Type 1 RIPs and type 2 RIPs A-chain share the same ‘RIP fold’, with the N-terminal domain consisting of β-strands and α-helices and C-terminal containing α-helices predominantly. The residues involved in the catalytic site (i.e., Glu176, Arg179 and Trp208, numbering according to the pokeweed antiviral protein (PAP) amino acid sequence) are structurally conserved [27].

**Figure 2:**
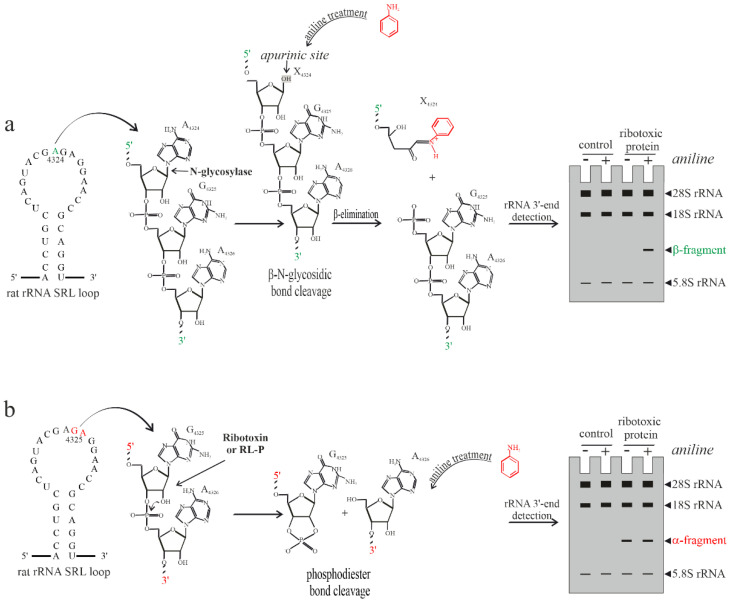
Schematic representation of diagnostic fragments release detection on gel electrophoresis after toxin-treated RNA from ribosomes. (**a**) RIP β–N-glycosidic bond cleavage; (**b**) ribotoxin or RL–P phosphodiester bond specific cleavage. SRL, sarcin ricin loop from rat rRNA.

rRNA N-glycosylase activity of plant RIPs was first documented by Endo and Tzurugi, who tested the action of ricin on the eukaryotic 28S rRNA from rats [28]. The authors described an effective method to detect the release of diagnostic β-fragment when the rRNA N-glycosylase-treated deadenylated RNA was incubated with acid aniline, followed by denaturing polyacrylamide gel electrophoresis (Figure 2a). The same protocol allows one to detect the presence of α-fragment diagnostic of both ribotoxin and RL-Ps action, which is visible on gel electrophoresis without acid aniline treatment (Figure 2b). In this case, the release of the specific rRNA 3′-end fragment is not dependent on aniline treatment, but is directly related to the rRNA endoribonuclease enzymatic action (i.e., ribotoxins or RL-Ps). Thus, the use of acid aniline is necessary only for the detection of β-fragments, released after abasic rRNA treatment with the aromatic amine.

Therefore, considering that to improve the fitness, the target of both parasites and their hosts is the ribosomes at the level of rRNA, Endo’s assay is the most used method to detect enzymes that are able to hydrolyze a specific phosphodiester bond (ribonucleases) or cleave a single N-glycosidic bond (rRNA N-glycosylases; plant ‘RIPs’). Indeed, the detection of α- or β-fragment release allows one to distinguish among these enzymes, although at the cellular level, the physiological action is the same (i.e., protein synthesis blocking).

In this framework, since the result of Endo’s assay is sometimes not reported, the enzymatic action of purified protein synthesis inhibitors is unclear. In particular, this ambiguity is often ascertained in the publications regarding the protein synthesis inhibitors isolated from mushrooms and other fungi.

Thus, in the present work, we report a careful revision of the available literature on protein synthesis inhibitors from mushrooms and other fungi, considering their correct classification on the basis of specific fragment (α- or β fragment) released after Endo’s assay, with or without acid aniline treatment. Moreover, other enzymes acting as inhibitors of protein synthesis in fungi have been reviewed in a separate section, considering their ability to inhibit protein synthesis by 50% (IC_50_) in a cell-free protein synthesis inhibitory system [29].

Currently, the Scopus database includes 144 or 48 documents using the following keywords: ‘ribosome inactivating fungi’ or ‘ribosome inactivating mushrooms’, respectively (https://www.scopus.com/; accessed on 10 April 2022). The screening revealed 13 different inhibitors of protein synthesis from fungi species, characterized from 1991 to 2021.

However, the diagnostic β-fragment was shown for only five toxins, while in other cases, rRNA N-glycosylase action was not confirmed or the presence of the α-fragment revealed the presence of specific ribonucleases, such as ribotoxins, from ascomycetes. Our search revealed that the literature is full of contradictory findings about the presence of rRNA N-glycosylases in mushrooms and other fungi.

In this framework, the Endo’s assay needs to be used to distinguish rRNA N-glycosylases by other enzymes that act as inhibitors of protein synthesis. This observation is supported by the extensive literature available on bolesatine from *Boletus satanas*. Indeed, a previous study included bolesatine in the group of protein synthesis inhibitors acting as a rRNA N-glycosylase analogous to plant RIPs, due to the ability of protein synthesis inhibitors in several in vitro systems and in vivo [30,31]. However, further research disproves the previous hypothesis by showing the absence of β-fragment release after bolesatine-treated RNA from rat liver ribosomes [32]. The bolesatine mechanism to inhibit protein synthesis was then elucidated by Ennamany et al. [33], which defined this toxin as a nucleoside triphosphate phosphatase, revealing that the mechanism whereby bolesatine affects protein synthesis involves nucleoside triphosphates hydrolysis (i.e., GTP), rather than directly elongation factors.

## 2. Fungal RIPs Able to Release the β-Fragment

### 2.1. Calcaelin from Calvatia caelata

Calcaelin is a RIP isolated from the fruiting bodies of edible basidiomycete mushroom *Calvatia caelata* (Bull.) Morgan, 1890 [34]. This protein shows a molecular mass of about 39 kDa, as determined by gel filtration and non-reducing SDS-PAGE analysis (two bands under reducing conditions; 19 and 20 kDa), while the N-terminal amino acid sequence obtained by automated Edman degradation is reported in Table 1.

Both protein yield and IC_50_ value in the cell-free translation system are reported in Table 2. The N-glycosylase activity was demonstrated by Endo’s assay after the detection of β-fragment in the presence of acid aniline on denaturing polyacrylamide gel electrophoresis. Calcaelin had neither antifungal nor antibacterial activity. It also did not exert hemagglutination. The protein was cytotoxic against human breast carcinoma cells and mouse splenocytes and displayed antimitogenic activity against mouse splenocytes.

### 2.2. Lyophyllin from Lyophyllum shimeji

Lyophyllin is a RIP isolated from the fruiting bodies of edible basidiomycete mushroom *Lyophyllum shimeji* (Kawam) Hongo, 1971 [35,36]. This protein resembles the M35 Zn^2+^-metallopeptidase extracellular domain of peptidyl-Lys metalloendopeptidases (MEPs; EC 3.4.24.20). On the other hand, although conserving the HEXXH+D+Y active pocket, lyophyllin is not able to cleave peptidyl–lysine bonds, as is the case with MEPs. This is due to an additional intrachain loop in lyophyllin’s amino acid sequences (positions 84–88), which may block the peptidase activity as previously demonstrated by the structural alignment of lyophyllin and a member of the M35 endopeptidase superfamily (GfMEP from *Grifola frondosa*; PDB code: 1g12) [35]. The protein shows a molecular mass of about 20 kDa, as determined by gel filtration and SDS-PAGE analysis [36]. Moreover, the protein sequence has been obtained by coupling MALDI ToF mass spectrometry (MS) and the screening of *L. shimeji* genome using the N-terminal achieved by Edman degradation [35], as shown in Table 1. Both protein yield and IC_50_ value in the cell-free translation system according to Lam and Ng, 2001 [36], are reported in Table 2. The N-glycosylase activity was demonstrated by Endo’s assay after the detection of β-fragments in the presence of acid aniline on denaturing polyacrylamide gel electrophoresis. Lyophyllin showed antifungal activity and HIV-1 reverse transcriptase inhibitory activities, which in turns employ deleterious effects on mouse embryonic development, but did not exert hemagglutination. The protein was cytotoxic against several cancer cell lines (i.e., Hela, HepG2, and JAR) in a dose-dependent manner and did not show ribonuclease activity.

### 2.3. Marmorin from Hypsizygus marmoreus

Marmorin is a RIP isolated from the fruiting bodies of edible basidiomycete mushroom *Hypsizygus marmoreus* (Peck) H.E. Bigelow, 1976 [37]. As determined by MALDI ToF MS, this RIP shows a molecular mass of 9,567 Da, which is close to the molecular weight limit for considering this protein as a peptide [40]. The N-terminal amino acid sequence obtained by automated Edman degradation is reported in Table 1. Both protein yield and IC_50_ value in the cell-free translation system are reported in Table 2. The N-glycosylase activity was demonstrated by Endo’s assay after the detection of β-fragments in the presence of acid aniline on denaturing polyacrylamide gel electrophoresis. Marmorin inhibited HIV-1 reverse transcriptase, but had neither antifungal nor hemagglutinating, mitogenic, protease, anti-mitogenic, RNase, trypsin inhibitory or nitric oxide-inducing activities. The protein was cytotoxic against hepato-carcinoma HepG2 and MCF-7 breast cancer cell lines, although it did not show ribonuclease activity.

### 2.4. Mucoricin from Rhizopus delemar

Mucoricin is a RIP isolated from the fungus *Rhizopus delemar* that belongs to the order mucorales (phylum zygomycota), the most important causal agent of mucormycosis [38]. The protein sequence has been obtained by LC-MS/MS identifying a protein of 17 kDa (Table 1). The IC_50_ values in the cell-free translation system are reported in Table 2. The N-glycosylase activity was demonstrated by Endo’s assay after the detection of β-fragments in the presence of acid aniline on denaturing polyacrylamide gel electrophoresis. The protein is cytotoxic against primary lung epithelial cells, A549 cells and human umbilical vein endothelial cells (HUVECs). Moreover, intravenously RIP injected into mice caused weight loss and mortality. Mucoricin has a role in mucormycosis pathogenesis, as confirmed by the effect of toxin expression downregulation (RNAi knockdown) on *R. delemar* virulence [38]. Finally, a model of pulmonary mucormycosis showed that although the toxin is not important for pathogenesis initiation, it is mainly implicated in disease lethality.

### 2.5. Volvarin from Volvariella valvacea

Volvarin is a RIP isolated from the fruiting bodies of edible basidiomycete mushroom *Volvariella valvacea* (Bull.) Singer, 1951 [39]. This protein shows a molecular mass of about 29 kDa, as determined by gel filtration and SDS-PAGE analysis, but there is no information on protein sequence. The IC_50_ value in the cell-free translation system is reported in Table 2. The N-glycosylase activity was demonstrated by Endo’s assay after the detection of β-fragments in the presence of acid aniline on denaturing polyacrylamide gel electrophoresis. It also exerted deoxyribonuclease activity on supercoiled SV-40 DNA and exhibited abortifacient activity, as previously reported for other type 1 RIPs from plants [41].

## 3. Other Ribotoxic Enzymes from Fungi Improperly Classified as N-Glycosylases

Considering the Endo’s assay as essential for RIP identification, in this section, we reviewed all the enzymes isolated from mushrooms and other fungi that are able to inactivate protein synthesis and are improperly described as N-glycosylases or RIPs. Indeed, in most of cases, N-glycosylase activity was not confirmed by the detection of β-fragments, diagnostic of N-glycosylase action. We also included ribotoxins improperly assimilated to the RIP family, since as shown by authors, the diagnostic fragment visible on denaturing polyacrylamide gel electrophoresis also without aniline treatment is clear evidence of phosphodiester bond specific cleavage (i.e., α-fragment) rather than deadenylation (β-fragment). On the other hand, protein synthesis is a very complex process, and the inhibitors do not always damage directly ribosomes by damaging the elongation factor-binding site (see bolesatine from *B. satanas*). Finally, evidence of these enzymes, found at the genomic level, has been included.

### 3.1. Bolesatine from Boletus satanas

Bolesatine is an inhibitor of protein synthesis isolated from the fruiting bodies of toxic basidiomycete mushroom *Rubroboletus satanas* (Lenz) Kuan Zhao et Zhu L. Yang, 2014 (basionym: *Boletus satanas* Lenz, 1831) [32,42,43,44]. This toxin is a basic glycoprotein (pI 8.3 ± 0.1) [30] with a molecular mass of about 63 kDa, as determined by SDS-PAGE analysis [42]. The N-terminal amino acid sequence obtained by automated Edman degradation is reported in Table 3. Both protein yield and IC_50_ value in cell-free translation system are reported in Table 4. In a previous study, the protein was proposed to be a possible RIP [30]. However, the Endo’s assay carried out by Krets et al. in 1992 disapproved the previous findings, due to the absence of β-fragments on gel electrophoresis, proving the absence of ribosome damaging [32]. Indeed, Ennamany et al. elucidated the mechanism of action of bolesatine, concluding that it inhibits protein synthesis by hydrolyzing nucleoside triphosphates (i.e., GTP) through nucleoside triphosphate phosphatase activity, rather than directly affecting the elongation factors binding site on the ribosomes [33]. Moreover, the glycoprotein bolesatine is considered a lectin and a potent mitogen for human T lymphocytes. Due to its immunomodulatory potential, the gastroenteritis related to *B. satanas* ingestion may be partially ascribed to bolesatine lectinic properties and its ability to activate immune responses [45].

### 3.2. Flammin from Flammulina velutipes

Flammin is an inhibitor of protein synthesis isolated from the fruiting bodies of edible basidiomycete mushroom *Flammulina velutipes* (Curtis) Singer, 1951 [46]. The protein molecular mass of about 30 kDa was determined by gel filtration and SDS-PAGE analysis. The N-terminal amino acid sequence obtained by automated Edman degradation is reported in Table 3. Both protein yield and IC_50_ value in cell-free translation system are reported in Table 4. The N-glycosylase activity was only mentioned; however, no denaturing polyacrylamide gel electrophoresis was shown to confirm the release of β-fragments after toxin-treated rRNA following aniline treatment. In this framework, it is impossible to definitively establish the N-glycosylase activity of flammin. Neither RNase or protease activities are attributed to this toxin [46].

### 3.3. Flammulin from Flammulina velutipes

Flammulin is an inhibitor of protein synthesis isolated from the fruiting bodies of edible basidiomycete mushroom *Flammulina velutipes* (Curtis) Singer, 1951 [47].

The molecular mass of the protein of about 40 kDa was determined by gel filtration and SDS-PAGE analysis. The N-terminal amino acid sequence obtained by automated Edman degradation is reported in Table 3. Both protein yield and IC_50_ value in cell-free translation system are reported in Table 4.

The N-glycosylase activity was only mentioned; however, no denaturing polyacrylamide gel electrophoresis was shown to confirm the release of β-fragment after toxin-treated rRNA following aniline treatment. In this framework, it is impossible to definitively establish the N-glycosylase activity of flammulin. No RNase activity is attributed to this toxin.

### 3.4. Hypsin from Hypsizygus marmoreus

Hypsin is an inhibitor of protein synthesis isolated from the fruiting bodies of edible basidiomycete mushroom *Hypsizygus marmoreus* (Peck) H.E. Bigelow, 1976 [48,52].

The protein exhibits a molecular mass of about 20 kDa, as determined by gel filtration and SDS-PAGE analysis. The N-terminal amino acid sequence obtained by automated Edman degradation is reported in Table 3. Both protein yield and IC_50_ value in cell-free translation system (very stable activity after trypsin treatment) are reported in Table 4. As occurred for the other protein synthesis inhibitors (i.e., flammin and velin from *F. velutipes*), the N-glycosylase activity was only mentioned, without showing Endo’s assay; thus, N-glycosylase activity could not be definitively elucidated. [48]. The enzyme displayed different properties, such as HIV-1 reverse transcriptase inhibitory activity, antifungal, antimitogenic and antiproliferative activities.

### 3.5. Pleuturegin from Pleurotus tuber-regium

Pleuturegin is an inhibitor of protein synthesis isolated from the sclerotia of edible basidiomycete mushroom *Pleurotus tuber-regium* (Fr.) Singer, 1951 [49]. The protein exhibited a molecular mass of about 38 kDa, as determined by gel filtration and SDS-PAGE analysis. The N-terminal amino acid sequence obtained by automated Edman degradation is reported in Table 3, while the IC_50_ value in the cell-free translation system is indicated in Table 4. As occurred for the other protein synthesis inhibitors (i.e., flammin and velin from *F. velutipes* or hypsin from *H. marmoreus*), the N-glycosylase activity was only mentioned, without showing Endo’s assay; thus, N-glycosylase activity could not be definitively established [49]. Finally, no ribonuclease activity was attributed to pleuturegin, excluding possible unspecific activity.

### 3.6. Tricholin from Trichoderma viride

Tricholin is an inhibitor of protein synthesis isolated from the culture medium of ascomycete fungus *Trichoderma viride* Pers., 1832 [50]. The protein is acidic and exhibited a molecular mass of 14.2 kDa, as determined by gel filtration and SDS-PAGE analysis. Since the N-terminal is blocked, no information on amino acid sequence by automated Edman degradation was achieved, while 630 nM tricholin completely inhibit protein synthesis in a cell-free system, as indicated in Table 4. Although tricholin has been associated to N-glycosylase enzyme analogues to plant RIPs, the Endo’s assay clearly indicated that this protein is a ribotoxin from ascomycetes, due to the release of diagnostic α-fragments, similarly to the prototype α-sarcin [50]. Moreover, this protein displayed ribonucleolytic activity on total RNA extracted from *E. coli*. Tricholin is clear evidence of the importance of using Endo’s assay to distinguish the type of protein synthesis inhibitor.

### 3.7. Velin from Flammulina velutipes

Velin is an inhibitor of protein synthesis isolated from the fruiting bodies of edible basidiomycete mushroom *Flammulina velutipes* (Curtis) Singer, 1951 [46]. The N-terminal amino acid sequence obtained by automated Edman degradation is reported in Table 3. Both protein yield and IC_50_ value in cell-free translation system are reported in Table 4. The N-glycosylase activity was only mentioned; however, no denaturing polyacrylamide gel electrophoresis was shown to confirm the release of β-fragments after toxin-treated rRNA following aniline treatment. In this framework, it is impossible to definitively establish the N-glycosylase activity of velin. The protein has neither RNase nor protease activity.

### 3.8. Velutin from Flammulina velutipes

Velutin is an inhibitor of protein synthesis isolated from the fruiting bodies of edible basidiomycete mushroom *Flammulina velutipes* (Curtis) Singer, 1951 [51]. The protein exhibits a molecular mass of 13.8 kDa, as determined by gel filtration and SDS-PAGE analysis. The N-terminal amino acid sequence obtained by automated Edman degradation is reported in Table 3. Both protein yield and IC_50_ value in cell-free translation system are reported in Table 4. In this case also, the N-glycosylase activity was only mentioned, without showing Endo’s assay; thus, N-glycosylase activity could not be definitively elucidated [51]. No ribonuclease activity was attributed to velutin, excluding possible unspecific activity. Moreover, the toxin is endowed with HIV-1 reverse transcriptase inhibitory activity.

### 3.9. Mushroom Type 2 Ribosome Inactivating Protein-like Genes

In 2017, Liu et al. cloned the gene encoding for a 32.33 kDa type 2 RIP-like protein named PuRIP (290 amino acid residues; pI 5.58) from sclerotia of the edible basidiomycete mushroom *Polyporus umbellatus*. The authors demonstrated that the expression of PuRIP occurred in all mushroom tissues and was upregulated by *Armillaria mellea* infection. Multiple sequence alignment showed that the PuRIP deduced amino acid sequence had conserved domains of ricin superfamily protein [53].

## 4. Isolation and Purification of Protein Synthesis Inhibitors from Mushrooms and Other Fungi

**Figure 3:**
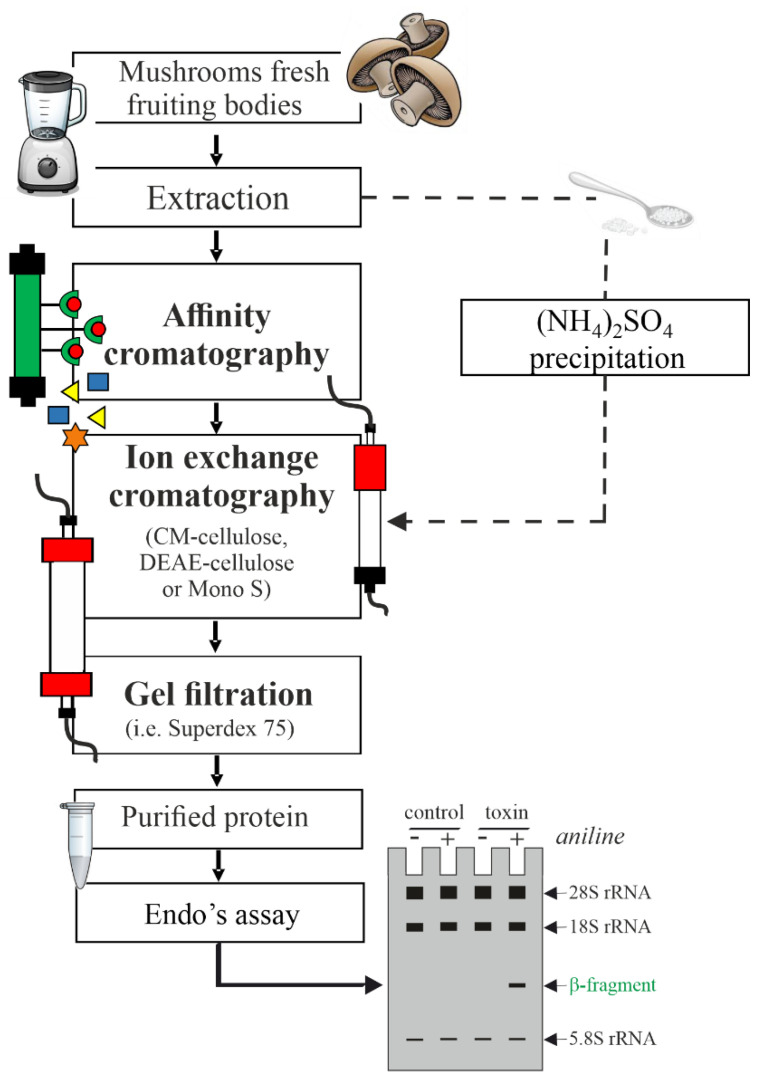
General workflow of isolation and purification of protein synthesis inhibitors that are able to release the β-fragment (N-glycosylases) from mushrooms and other fungi.

Despite the higher variability in molecular masses and N-terminal sequences, the procedures for isolation and purification of N-glycosylases (analogues to plant RIPs) from mushrooms and other fungi are similar (Figure 3). A procedure usually used for the isolation of type 1 RIPs from plants was carried out for the obtainment of both fungal RIPs and protein synthesis inhibitors. As a starting material, mushroom fresh fruiting bodies were homogenized in neutral or acid buffer (e.g., 10 mM Tris•Cl pH 7.2 or 10 mM NH_4_OAc, pH 4.6) and the crude extract was subjected to affi-gel blue gel chromatography or ammonium sulphate precipitation. Moreover, one or more steps of ion exchange chromatography by DEAE-cellulose, Mono S or CM-cellulose column can be employed, performing the elution by increasing the ionic strength and/or pH. In some cases, ion exchange chromatography can precede or replace affi-gel blue gel chromatography or precipitation (e.g., lyophyllin and marmorin purification [36,37]). Finally, gel filtration by Superdex 75 was usually applied as last step, allowing one to also obtain information on protein molecular mass. However, for the isolation and purification of some N-glycosylases (i.e., mucoricin from *R. delemar*), fungal spores were cultured in YPD medium and the fungal mat was ground in liquid nitrogen, extracted with sterile H_2_O, concentrated and subjected to gel filtration. The active fractions (human A549 cell line damaging) with a molecular weight of 10–30 kDa were then subjected to 3D chromatographic separations and the peak with higher activity was purified by RP-HPLC [38].

## 5. Sequence Alignment and Phylogenetic Analysis

Despite the fact that fungal genomic resources are increasing due to the expansion of fungal genome databases, deciphering the complexity of fungal genomes remains a significant challenge for researchers. Moreover, our research has highlighted that little information on amino acid sequences of ascertain fungal protein synthesis inhibitors that are able to release the β-fragment is available in the literature. In this scenario, we used the available complete amino acid sequences of lyophyllin and mucoricin from basidiomycota or mucoromycota division, respectively, as query to identify fungal homologous sequences by using the FASTA search against UniProt Knowledgebase database (https://www.ebi.ac.uk/Tools/sss/fasta/; accessed on 20 April 2022). In particular, the similarity search, using as query the lyophyllin domain, displayed similar amino acid sequences in MEPs. On the other hand, the similarity search, using the amino acid sequence of mucoricin as query, shows protein sequence similarity to ricin B-type lectin domain-containing proteins, carbohydrate-binding proteins, pyridoxal 5′-phosphate synthases or uncharacterized proteins, although the protein is monomeric and possesses N-glycosylase activity. For each query, we chose the most similar amino acid sequences from the top 50 hits, considering their distribution in different fungi that belong to basidiomycota or mucoromycota division.

**Figure 4:**
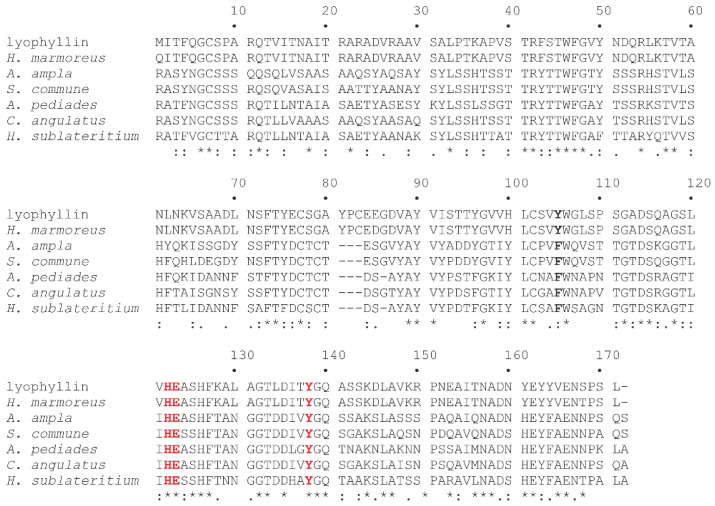
Alignment of the amino acid sequences of lyophyllin from *L. shimeji* with lyophyllin-like proteins performed using the ClustalW tool (https://embnet.vital-it.ch/software/ClustalW.html; accessed on 25 April 2022) with default parameters. Identical residues (*), conserved substitutions (:), and semi-conserved substitutions (.) are reported. For protein information, see Supplementary Table S1. Conserved amino acid residues necessary for N-glycosylase activity are highlighted in red, while the amino acid residue important for stacking the target adenine ring is highlighted in bold.

The alignment between lyophyllin and other MEPs that belong to basidiomycetes is shown in Figure 4. All the domains retrieved contain His122, Glu123 and Tyr138 conserved amino acid residues, which are necessary for N-glycosylase activity, as demonstrated by site-direct mutagenesis [35]. On the other hand, Tyr105, which is important for stacking the target adenine ring, in some cases is replaced by a phenylalanyl residue.

**Figure 5:**
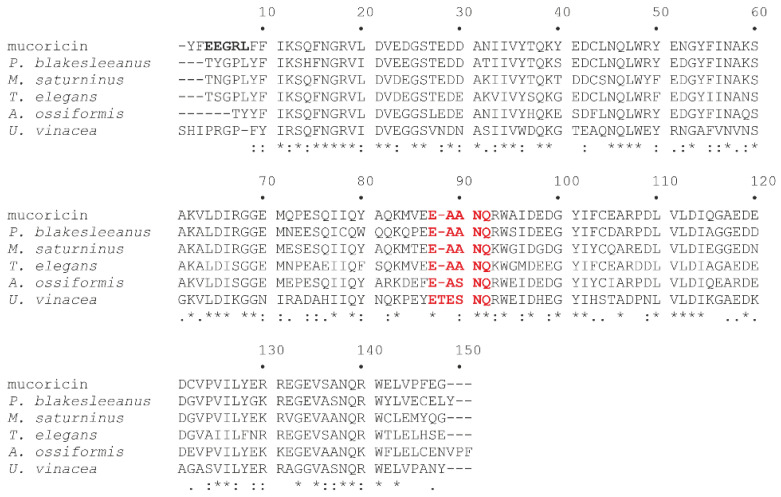
Alignment of the amino acid sequences of mucoricin from *R. delemar* with mucoricin-like proteins performed using the ClustalW tool (https://embnet.vital-it.ch/software/ClustalW.html; accessed on 25 April 2022) with default parameters. Identical residues (*), conserved substitutions (:), and semi-conserved substitutions (.) are reported. For protein information, see Supplementary Table S1. The two motifs (EEGRL and EAANQ) hypothesized to be responsible for N-glycosylase activity are highlighted in bold and red, respectively.

As shown by molecular docking, the indicated key amino acid residues important for lyophyllin N-glycosylase activity resemble E160, R163, Y70, and Y111 of trichosanthin type 1 plant RIP from *Trichosanthes kirilowii* [35]. A different scenario is presented considering the alignment between mucoricin and other retrieved similar proteins that belong to mucorales, as shown in Figure 5. Indeed, Soliman et al. hypothesized that mucoricin has two motifs responsible for N-glycosylase activity. The first one is the EEGRL motif at the N-terminal domain, corresponding to the catalytic site (EAARF) of ricin A-chain, in which Glu and Arg amino acid residues implicated in N-glycosylase activity are conserved [38].

The second one is the EAANQ motif, which resembles the EAARF motif of the ricin B-chain, in which Glu amino acid residue is conserved, while Arg is replaced by Asn, an amino acid residue that shares weakly similar properties with Arg. This substitution could explain the weaker N-glycosylase activity of mucoricin in comparison to ricin (~800-fold more active) [38]. Considering the two motifs, EEGRL is not present in the other mucoricin-like sequences (Figure 5), while the EAANQ is conserved in all homologous amino acid sequences, except for the *U. vinacea* protein, although the key amino acid residues (i.e., Glu and Asn) of the EAANQ motif are conserved in all sequences. Therefore, in this case, we are not sure whether the similar amino acid sequences found in the protein database are endowed with N-glycosilase activity.

**Figure 6:**
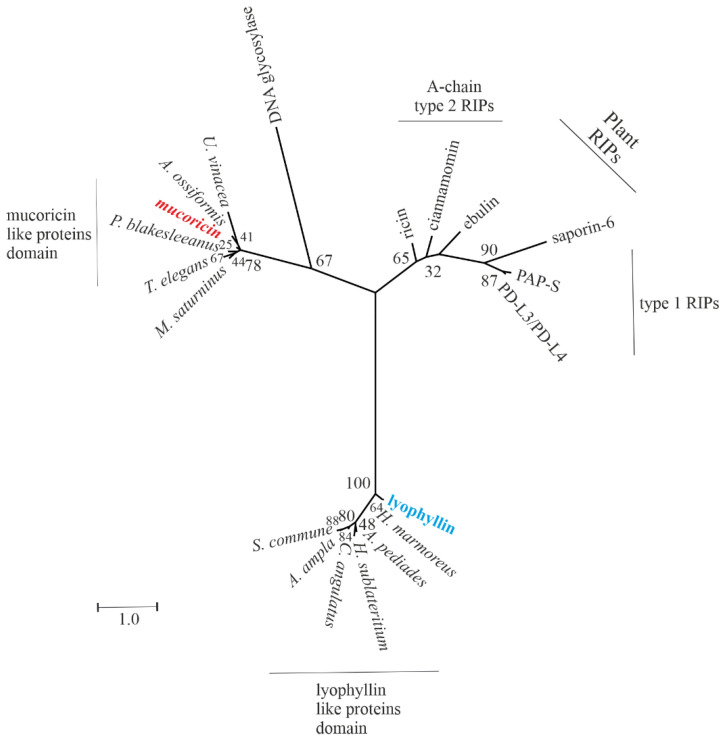
Radial cladogram showing the diversity of N-glycosylases from fungi and analogous N-glycosylases from plants. The evolutionary history was inferred using the maximum likelihood method and JTT matrix-based model. The tree with the highest log likelihood (−7380.45) is shown. The percentage of trees in which the associated taxa clustered together is shown next to the branches. Initial tree(s) for the heuristic search were obtained automatically by applying neighbor-join and BioNJ algorithms to a matrix of pairwise distances estimated using the JTT model, and then selecting the topology with superior log likelihood value. The tree is drawn to scale, with branch lengths measured in the number of substitutions per site. This analysis involved 20 amino acid sequences. Lyophyllin from *L. shimeji* and mucoricin from *R. delemar* are highlighted in blue and red, respectively. For further information, see Supplementary Table S1. There were 347 positions in the final dataset. Evolutionary analyses were conducted in MEGA11.

Finally, the analysis of the phylogenetic relationship was performed after the alignment of the fungal homologous sequences by using the ClustalW tool available online (https://embnet.vital-it.ch/software/ClustalW.html; accessed on 25 April 2022). Moreover, for the phylogenetic analysis, six of the most representative plant RIPs (three sequences for type 1 RIPs and three for A-chain type 2 RIPs) were included. RIP amino acid sequences from plants were obtained from the UniProt database (http://www.uniprot.org/; accessed on 25 April 2022). The accession numbers of the RIPs used are reported in Supplementary Table S1. The evolutionary history was obtained by Molecular Evolutionary Genetics Analysis (MEGA) software, version 11 https://www.megasoftware.net/ (accessed on 25 April 2022) and the phylogenetic tree was constructed by employing the maximum likelihood method, based on the JTT matrix-based model option [54]. Phylogeny was tested by 500 bootstrap replications [55]. The sequence of uracil-DNA-glycosylase from *Artemisia annua* was used as an outgroup. The tree designed as a radial cladogram shows three main separated groups (Figure 6). Group 1 contains plant RIPs, further divided into two distinct subgroups, type 1 and A-chain type 2 RIPs, respectively. On the other hand, group 2 and 3 contain mucoricin and mucoricin-like proteins or lyophyllin and lyophyllin-like proteins, respectively.

Our results display the large difference between plant and fungal N-glycosylases, and the two hypothetical groups retrieved to date in fungi. This divergence is consistent with the different active site retrieved or hypothesized for lyophyllin-like proteins (site-direct mutagenesis) or mucoricin-like proteins (bioinformatics approach), respectively. Considering the above, in our opinion, the N-glycosylases found in fungi that are able to inhibit protein synthesis are analogous to the N-glycosylases found in plants (plant RIPs), due to the different structural features.

## 6. Concluding Remarks

A search in the literature revealed that 13 fungal enzymes purified and characterized from mushrooms and other fungi are able to inhibit protein synthesis and their activity was associated to that of analogue RIPs from plants. However, after careful revision, we found that rRNA N-glycosylase activity was only uncertain for five enzymes, which were calcaelin from *C. caelata*, lyophyllin from *L. shimeji*, marmorin from *H. marmoreus*, mucoricin from *R. delemar* and volvarin from *V. valvacea*. Therefore, we used the Endo’s assay to detect enzymes that hydrolyze a specific phosphodiester bond (ribonucleases) or cleave a single N-glycosidic bond (rRNA N-glycosylases; plant ‘RIPs’). Indeed, the detection of α- or β-fragment release, with and without acid aniline treatment, allows one to distinguish between the two different enzymatic activities, although at the cellular level, the physiological action is the same (i.e., protein synthesis blocking).

In addition, we used the structural information available on fungal N-glycosylases to perform a phylogenetic analysis, revealing the difference between plant and fungal N-glycosylases, showing the presence of two hypothetical groups retrieved to date in fungi with structural differences, which can be considered analogues to rRNA N-glycosylases from plants.

In this scenario, further studies will be necessary in order to clarify the structural features and the role of these enzymes (i.e., fungal N-glycosylases) in mushrooms and other fungi. It is also necessary to consider them from an evolutionary point of view as a novel example of analogue enzymes that share the same target but likely with different catalytic mechanisms (convergent evolution).

## Figures and Tables

**Table 1:** N-terminal amino acid sequence of fungal RIPs reported in the literature. ‘NCBI: txid’ refers to the taxonomy ID of mushrooms reported in NCBI taxonomy browser.

Enzyme	Source	M*r* (Da)	N Terminal Sequence	Taxonomy NCBI: Txid	Ref.
Calcaelin	*Calvatia caelata*	39,000	_1_ ANPIYNIDAF RV _12_	1916349	[34]
Lyophyllin	*Lyophyllum shimeji*	20,000	_1_ ITFQGCSPAR QTVITNAITR ARADVRAAVS _30_ *	47721	[35,36]
Marmorin	*Hypsizygus marmoreus*	9567	_1_ AEGTLLGSRA TCESGNSMY _19_	39966	[37]
Mucoricin	*Rhizopus delemar*	17,000	_1_ YFEEGRLFFI KSQFNGRVLD VEDGSTEDDA _30_ *	936053	[38]
Volvarin	*Volvariella valvacea*	29,000	n.d.	36659	[39]

n.d. not determined. * First 30 amino acid residues. For the complete amino acid sequence, see the reference

**Table 2:** Protein yield and IC_50_ values of fungal RIPs. Protein synthesis inhibitory activity assay was based on cell-free protein synthesis system. Luciferase mRNA (luminescence measured by the luminometer) or incorporation of radiolabelled amino acid into protein ([^35^S]-methionine or [^3^H]-leucine incorporation measured by liquid scintillation counting) was used as the protein synthesis reporter into the rabbit reticulocyte lysate translation system.

Enzyme	Source	Protein Yield (mg/kg)	IC_50_ Value (nM)	Ref.
Calcaelin	*Calvatia caelata*	10.0	4.0	[34]
Lyophyllin	*Lyophyllum shimeji*	1.66	1.0	[35,36]
Marmorin	*Hypsizygus marmoreus*	2.2	0.7	[37]
Mucoricin	*Rhizopus delemar*	n.r.	17	[38]
Volvarin	*Volvariella valvacea*	n.r.	0.5	[39]

n.r. not reported.

**Table 3:** N-terminal amino acid sequence of protein synthesis inhibitors from mushrooms and other fungi reported in the literature. ‘NCBI: txid’ refers to the taxonomy ID of mushrooms reported in NCBI taxonomy browser.

Enzyme	Source	M*r* (Da)	N Terminal Sequence	Taxonomy NCBI: Txid	Ref.
Bolesatine	*Boletus satanas*	63,000	_1_ TWRIYLNNQT VKLALLLPNG _20_	5370	[32]
Flammin	*Flammulina velutipes*	30,000	_1_ SPVIPANTFV AFRLYEVGIV PA _22_	38945	[46]
Flammulin	*Flammulina velutipes*	40,000	_1_ APSHFSHPGV LADRAQIDFI XGKVNEGAEP WXSAYN _36_	38945	[47]
Hypsin	*Flammulina velutipes*	20,000	_1_ ITFQGDLDAR QQVITNADTR RKRDVRAAVR _30_	38945	[48]
Pleuturegin	*Pleurotus tuber-regium*	38,000	_1_ ARTQPGNIAP VGDFTLYPNA PRQGHIVA _28_	716892	[49]
Tricholin	*Trichoderma viride*	14,200	n.d.	5547	[50]
Velin	*Flammulina velutipes*	19,000	_1_ SGSPLTQAQA EALLKPQGLA YSSGGNT _27_	38945	[46]
Velutin	*Flammulina velutipes*	13,800	_1_ XHPDLFXXRP DNTASPKFED PRLNP _25_	38945	[51]

n.d. not determined.

**Table 4:** Protein yield and IC_50_ values of protein synthesis inhibitors from mushrooms and other fungi. Protein synthesis inhibitory activity assay was based on cell-free protein synthesis system. Incorporation of radiolabelled amino acid into protein ([^35^S]-methionine, [^3^H]-leucine or [^14^C] incorporation measured by liquid scintillation counting) was used as the protein synthesis reporter into the rabbit reticulocyte lysate translation system.

Enzyme	Source	Protein Yield (mg/kg)	IC_50_ Value (nM)	Ref.
Bolesatine	*Boletus satanas*	0.23 *	33	[32]
Flammin	*Flammulina velutipes*	1.28	1.4	[46]
Flammulin	*Flammulina velutipes*	0.44	0.25	[47]
Hypsin	*Flammulina velutipes*	10.0	7.0	[48]
Pleuturegin	*Pleurotus tuber-regium*	n.r.	0.5	[49]
Tricholin	*Trichoderma viride*	n.r.	630 ^#^	[50]
Velin	*Flammulina velutipes*	1.1	2.5	[46]
Velutin	*Flammulina velutipes*	16.28	0.29	[51]

n.r. not reported; * percentage expressed in total proteins, as reported in Kretz et al., 1989 [43]; ^#^ 100% of protein synthesis inhibition.

## Data Availability

The data presented in this study are available in this article.

## Supplementary Materials

The following supporting information can be downloaded at: https://www.mdpi.com/article/10.3390/toxins14060403/s1. Table S1: Accession numbers, species and common names of ribosome inactivating proteins sequences used in this work for primary structure analyses.

## Abbreviations

MEPs	peptidyl-Lys metalloendopeptidases
MS	mass spectrometry
RIPs	ribosome inactivating proteins
RL-Ps	ribotoxin-like proteins
SRL	sarcin ricin loop
