# Supplementary material for: Ribotoxic Proteins, Known as Inhibitors of Protein Synthesis, from Mushrooms and Other Fungi According to Endo’s Fragment Detection
Source: Toxins (Basel). 2022 Jun 13;14(6):403. doi: 10.3390/toxins14060403 (PMC9227437; doi:10.3390/toxins14060403)
Supplement: Supplementary file 1 [file toxins-14-00403-s001.zip › toxins-1754300-supplementary.pdf]

Table S1. Accession numbers, species and common names of ribosome inactivating proteins sequences used in this work for primary structure analyses.

N	Database : ID	Protein	Organism	Notes
1	A0A369K8V5	peptidyl-Lys metalloendopeptidase lyophyllin	*Hypsizygus marmoreus*	lyophyllin-like 1
2	A0A550CMU2	aspzincin_M35 domain-containing protein	*Auriculariopsis ampla*	lyophyllin-like 2
3	D8Q666	aspzincin_M35 domain-containing protein	*Schizophyllum commune*	lyophyllin-like 3
4	A0A8H4VKN6	uncharacterized protein	*Agrocybe pediades*	lyophyllin-like 4
5	A0A8H5B176	uncharacterized protein	*Coprinellus angulatus*	lyophyllin-like 5
6	A0A0D2PCR9	aspzincin_M35 domain-containing protein	*Hypholoma sublateritium*	lyophyllin-like 6
7	I1C083	mucoricin	*Rhizopus delemar*	
8	A0A167P735	carbohydrate-binding module family 13 protein	*Phycomyces blakesleeanus*	mucoricin-like 1
9	A0A8H7R6A7	uncharacterized protein	*Mucor saturninus*	mucoricin-like 2
10	A0A8H7VWX2	uncharacterized protein	*Thamnidium elegans*	mucoricin-like 3
11	A0A8H7ENF4	uncharacterized protein	*Apophysomyces ossiformis*	mucoricin-like 4
12	A0A8H7PQU0	uncharacterized protein	*Umbelopsis vinacea*	mucoricin-like 5
13	P23339.1	PAP-S	*Phytolacca americana*	plant type 1 RIP
14	P20656.2	saporin-6	*Saponaria officinalis*	plant type 1 RIP
15	P84854.2	PD-L3/PD-L4	*Phytolacca dioica*	plant type 1 RIP
16	AAK82460.1	cinnamomin	*Cinnamomum camphora*	plant type 2 RIP; chain-A
17	1HWM_A	ebulin-1	*Sambucus ebulus*	plant type 2 RIP; chain-A
18	1BR5_1	ricin A chain	*Ricinus communis*	plant type 2 RIP; chain-A
19	PWA35173.1	uracil-DNA-glycosylase	*Artemisia annua*	outgroup
